# The association of familial factors with drug treatment compliance in psychotic disorders in adolescents and young adults: a follow-up study of former adolescent inpatients

**DOI:** 10.1192/j.eurpsy.2025.766

**Published:** 2025-08-26

**Authors:** S. Eskelinen

**Affiliations:** 1 Research Unit of Clinical Medicine, Psychiatry, University of Oulu; 2 Oulu university hospital, Oulu, Finland

## Abstract

**Introduction:**

Schizophrenia and other psychotic disorders have a high social and economic cost. Antipsychotic drugs are the main approach to schizophrenia treatment. Drug adherence can be assessed with the Medication Possession Ratio (MPR), which means the days covered by the drugs purchased / 1 year.

**Objectives:**

The aim of this study was to investigate the effect of primary family factors and adverse childhood experiences (ACEs) on antipsychotic MPR among patients with schizophrenia spectrum disorder (SSD). Furthermore, we analyzed long-acting injectable antipsychotics (LAIs) and mood stabilizers separately.

**Methods:**

We had access to a database of former adolescent psychiatric inpatients (n=508) treated during the years 2001-2006 in Oulu university hospital, Finland. Participants were followed for SSD diagnosis via National care register for healthcare (CRHC) and physician-prescribed antipsychotic drug purchases via Social Insurance Institute (SII) register up to June 2023.

**Results:**

The participants using clozapine (OR 5.26, 95%CI 1.79-15.39) or mood stabilizers (OR 5.34, 95%CI 1.37-20.83) were significantly more likely to have MPR > 80% compared to participants using other antipsychotics. Sibling position, the size of primary family or ACEs did not associate with MPR.

**Conclusions:**

Clozapine and mood stabilizers increased the likelihood of higher antipsychotic MPRs among former adolescent psychiatric inpatients having SSD.

**Disclosure of Interest:**

None Declared

